# Comparative transcriptome analysis two genotypes of *Acer truncatum* Bunge seeds reveals candidate genes that influences seed VLCFAs accumulation

**DOI:** 10.1038/s41598-018-33999-3

**Published:** 2018-10-19

**Authors:** Rongkai Wang, Pei Liu, Jinshuan Fan, Lingli Li

**Affiliations:** College of Forestry, Northwest A&F University, Yangling, 712100 China

## Abstract

The *Acer truncatum* Bunge is a particular widespread forest tree species in northern China. VLCFAs are important to eukaryotes survival and play diverse roles throughout the development. So far, there are reports that the *Acer truncatum* seeds fatty acid (FA) rich in VLCFAs, but little is known about the physiological mechanism responsible for the biosynthesis. A total of approximately 37.07 Gbp was generated, it was comprehensive enough to determine the majority of the regulation VLCFAs biosynthesis genes. The 97,053 different unigenes were assembled and identified, and large numbers of EST-SSRs were determined. The expression profiles of crucial genes (*KCS*, *KCR*, *HCD* and *ECR*) involved in VLCFAs elongation of fatty acids were also studied. To our knowledge, the present study provides the first comprehensive of the transcriptome of *Acer truncatum* seeds. This transcriptome dataset have been made publicly available NCBI, we believe that it may provide new resource for future high-throughput gene expression of *Acer truncatum* seeds growth and development and will provide theoretical basic information for improving the yield of VLCFAs, especially nervonic acid.

## Introduction

The *Acer truncatum* Bunge, a particular widespread forest tree species in northern China, Japan and Korea, and is also found in Europe and Northern America^1,2^. The seeds fatty acid (FA) rich in nervonic acid (24:1; *cis*-tetracos-15-*en*oic acid) and it has been officially admitted as edible oil by the Ministry of Health of China^3^. Nervonic acid is a very long chain fatty acids (VLCFAs). VLCFAs are fatty acids with an acyl chain of 20–30 carbons and longer^4^. The chain lenth, the type of polar head, the degree of unsaturation and the associated lipids provide the structural and functional diversity of these fatty acids. VLCFAs are important to eukaryotes survival and play diverse roles throughout the growth and development^5^. In addition, VLCFAs are important feedstocks for industrial, pharmaceutical and nutraceutical applicatiom^6,7^. Vegetable oil, as neat fuel, has been to the main source of VLCFAs; therefore, the increase of VLCFA contents in seeds has become an important target for oilseed enhancement.

VLCFAs are synthesized by a microsomal fatty acid extension (FAE) system. Specifically, the C18 fatty acid is first synthesized using the *de novo* fatty acid synthesis pathway of plastids, and the C2 portion of malonyl-coenzyme A (CoA) is sequentially added to the previously synthesized C18 fatty acids^8,9^. Analogous to FAS, the FAE is a membrane-bound fatty acid elongation complex, which involves 4 enzymatic reactions: condensation of C18-CoA with malonyl CoA to form a ketoacyl-CoA by ketoacyl-CoA synthase (KCS), reduction of ketoacyl-CoA to a hydroxyacyl-CoA by ketoacyl-CoA reductase (KCR), dehydration of hydroxyacyl-CoA to a enoyl-CoA by hydroxyacyl-CoA dehydratase (HCD), and reduction of enoyl-CoA by enoyl-CoA reductase (ECR)^10^. The KCS is thought to be the rate-limiting enzyme for the elongation of long-chain fatty acids, since it determines the substrate and tissue specificity of long-chain fatty acid elongation. In addition, regulating the expression of the *KCS* gene affects the final contents of VLCFAs. In contrast, the other 3 enzymes have no substrate specificity and tissue specificity for VLCFA biosynthesis^11–19^.

Recently, RNA-Seq has become a very effective and powerful technology in generating comprehensive transcriptome dataset^20^. Studies in the past have indicated the rapid identification and profiling of differentially expressed genes by *de novo* transcriptome sequencing in some oil seeds, such as flax, castor bean, olive, peanut, sea buckthorn, tree peony and *Camellia oleifera*^21-27^. So far, there are reports that the *Acer truncatum* seeds FA rich in VLCFAs^3^, but little is known about the physiological mechanism responsible for VLCFAs biosynthesis.

In this study, we analyzed the transcriptome of *Acer truncatum* using high-throughput Illumina sequencing technology. In total, more than 37.07 Gbp was generated, it was comprehensive enough to determine the majority of the regulation VLCFAs biosynthesis genes. The 127,791 different transcripts and 97,053 unigenes were assembled and identified, and large numbers of EST-SSRs were determined. To our knowledge, this study provides the first comprehensive of the transcriptome of *Acer truncatum* seeds. This transcriptome dataset have been made publicly available NCBI, we believe that it may provide new resource for future research on bioengineering breeding and will provide theoretical basic information for improving the yield of VLCFAs.

## Results and Discussion

### FA profiling in different genotypes of *Acer truncatum* seeds

Based on the analysis of thirty-six genotypes *Acer truncatum* seeds FA, we found that different genotypes seeds showed different FA content and composition. There mainly were six kinds of VLCFAs in *Acer truncatum*, namely arachidic acid (C20:0), eicosenoic acid (C20:1), behenic acid (C22:0), erucic acid (C22:1), lignoceric acid (C24:0), and nervonic acid (C24:1). Although the accumulation of major VLCFAs varied in different genotypes seeds, the three dominant components of VLCFAs composed of eicosenoic acid (C20:1), erucic acid (C22:1) and nervonic acid (C24:1) (Tables S1 and S2).

**Figure 1:**
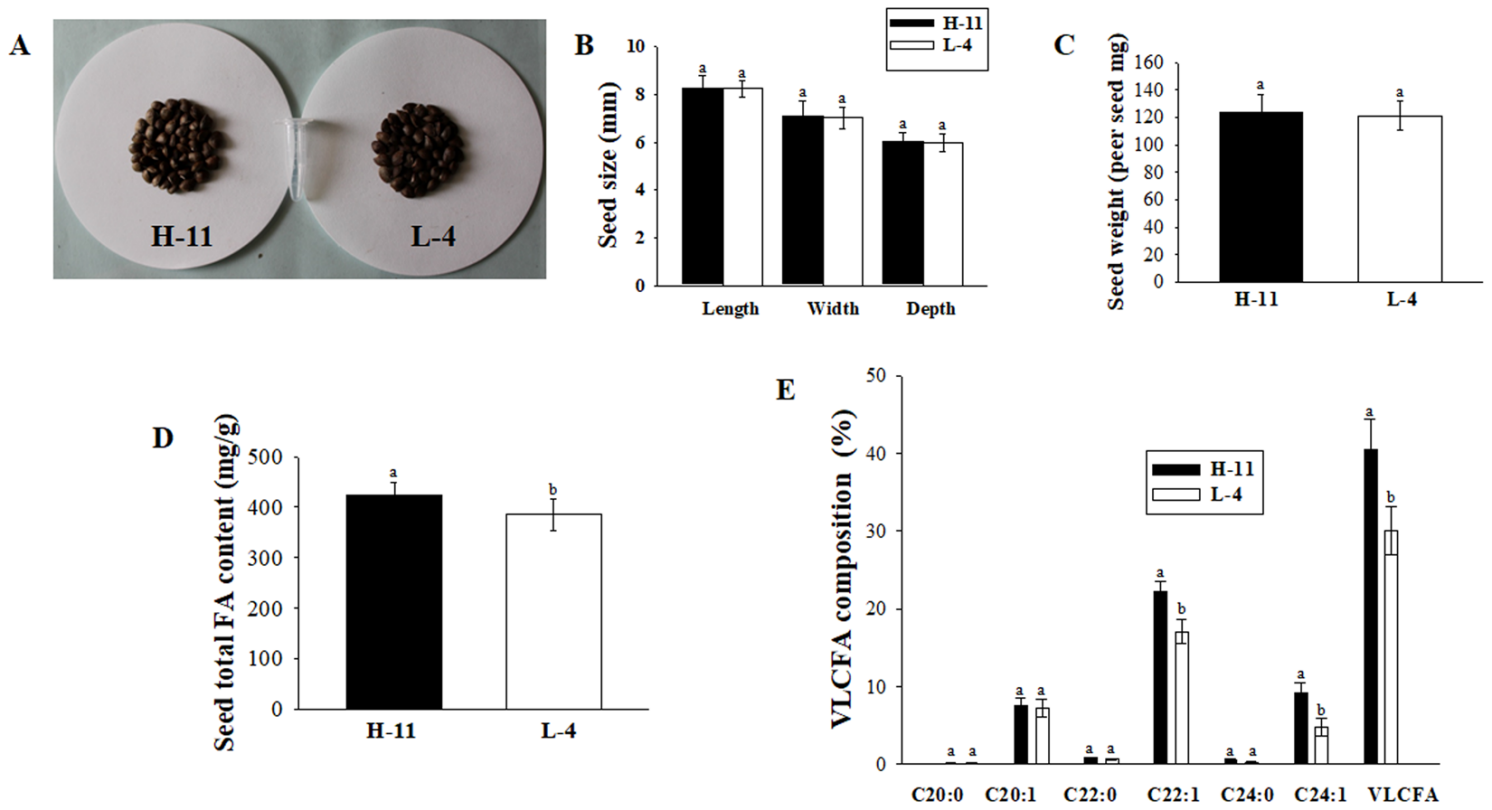
Characterization of two genotypes of *Acer truncatum* seeds. (**A**) The seeds randomly selected from the H-11 and L-4 plants. (**B**) Quantitative comparisons of seed size between the H-11 and L-4 plants. (**C**) Quantitative comparison of dry weight of seeds between the H-11 and L-4 plants. (**D**) Comparison of seeds total FA content between the H-11 and L-4 plants. (**E**) Comparison of contents of major seeds VLCFAs compositions between the H-11 and L-4 plants. Error bars are standard errors of the mean from three technical replicates. The different letters indicates significant differences at P < 0.05 (Student’s t-test).

The results showed that the highest content of VLCFAs genotype was H-11, and the lowest genotype was L-4. Additionally, there were higher content of Nervonic acid (C24:1) in H-11 seeds. However, no obvious differences were observed in several seed morphological traits including seed seed size and dry weight between seeds of H-11 and L-4 plants seeds (Fig. 1 and Table S2). To investigate the biological function on seed VLCFAs accumulation, we performed transcriptome sequencing of these two genotype seeds.

### Illumina sequencing and de novo assembly

**Figure 2:**
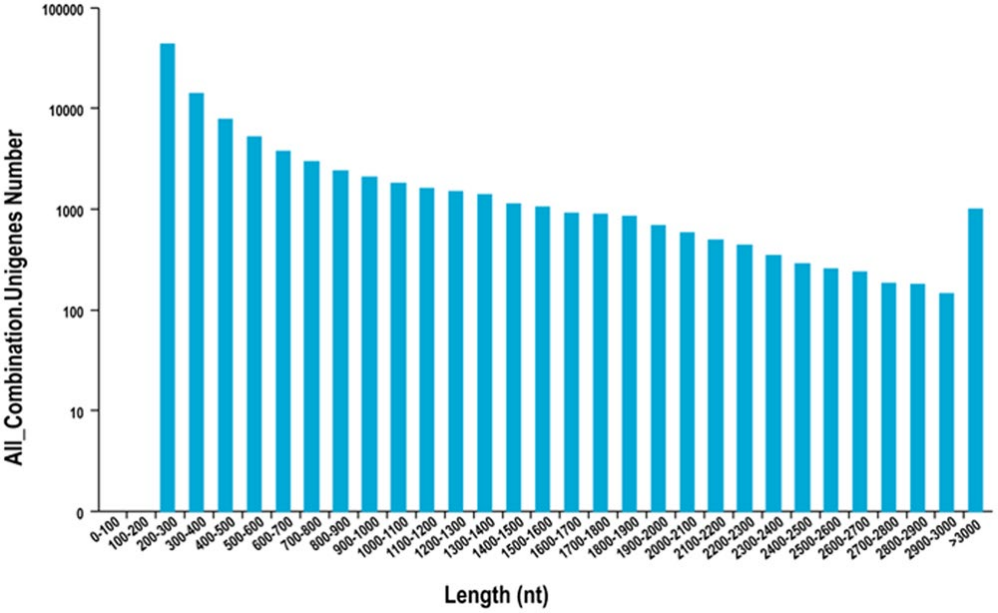
Length distribution of *Acer truncatum* transcripts.

The total of the *Acer truncatum* seeds mRNA was isolated from a single plant (H-11 or L-4). The sequencing raw data through rigorous quality assessment and data filtering, about 6.23 Gb, 6.21 Gb and 6.08 Gb, as well as 6.13 Gb, 6.21 Gb and 6.21 Gb for H-11 and L-4, respectively. It is presented in Table 1. The high quality sequencing reads has been deposited in the NCBI. Using the Trinity software program, the high quality sequencing reads were *de novo* assembled^28^, which produced 127,791 transcripts with an N50 length of 1,122 bp and a mean length of 686.76 bp. The distribution of the transcripts are depicted in Fig. S1 and Table S3. These transcripts were further analyzed for cluster and assembly. We have obtained 97,053 unigenes with an N50 length of 938 bp and a mean length of 598.19 bp for further analysis. It is shown in Fig. 2 and Table 2.

**Table 1:** Summary of transcriptome sequencing for two genotypes of *Acer truncatum* H-11 and L-4 seeds.

Sample	Read length	No. of reads	Data (bp)	GC%	Q30 (%)
H-11-1	150 + 150	20,777,973	6,233,391,900	53.27	88.90
H-11-2	150 + 150	20,689,010	6,206,703,000	52.24	89.49
H-11-3	150 + 150	20,278,336	6,083,500,800	52.34	89.18
L-4-1	150 + 150	20,417,206	6,125,161,800	49.59	89.52
L-4-2	150 + 150	20,712,428	6,213,728,400	51.96	90.06
L-4-3	150 + 150	20,705,377	6,211,613,100	50.44	89.61

**Table 2:** Overview of the *Acer truncatum* transcriptome unigenes assembly.

Unigenes length	Total number	Percentage
200-300	43,021	44.33%
300–500	21,857	22.52%
500–1000	16,338	16.83%
1000–2000	11,706	12.06%
2000+	4,131	4.26%
Total number	97,053	
Total length	58,055,717	
N50 length	938	
Mean length	598.19	

### Functional annotation of all unigenes

**Figure 3:**
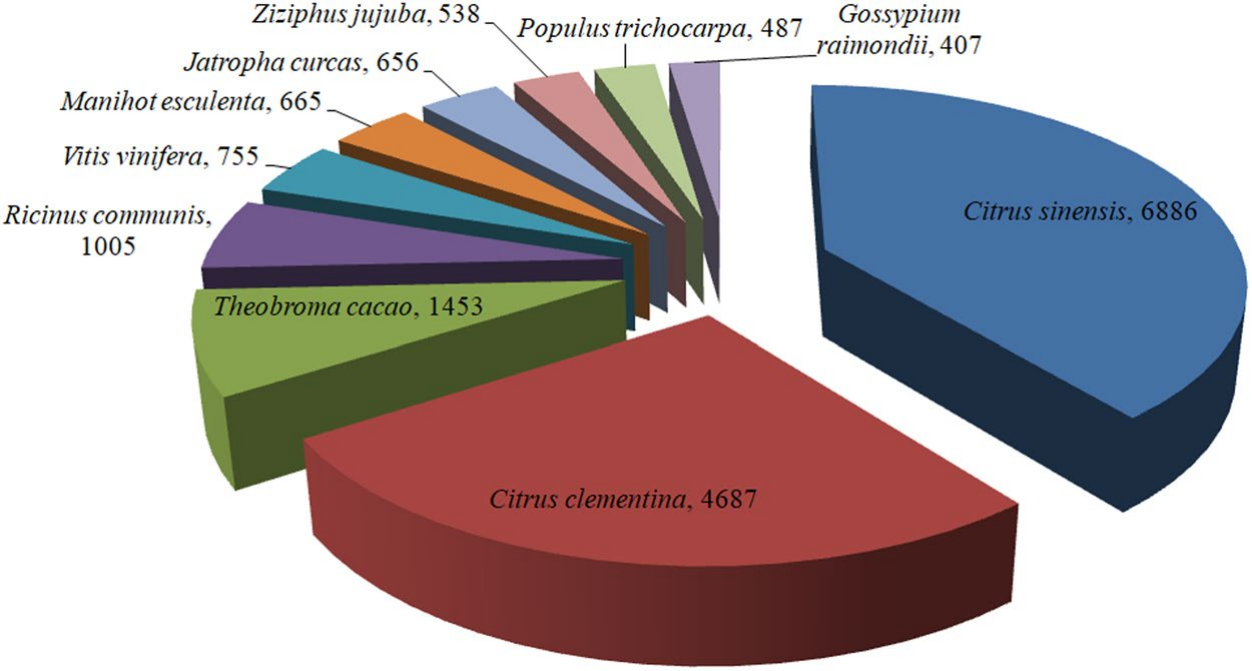
Species distribution of the top BLAST hits in the NR database.

The assembled unigenes sequences were annotated based on the following databases: NR, SWISS-PROT, GO, COG and KEGG. An overview of functional annotation in Table 3. We used BLASTX to similarity analysis and compared against the NR database. In these assembled unigenes, 71,014 (73.57%) unigenes had significant matches, and about 30% unigenes were showed no significant matches. Previous studies have shown that sequencing of cDNA libraries does not significant hits sequences is about 25% to 35%^29–31^. Among a wide range of plants with protein sequences, the *Acer truncatum* assembled unigenes had the highest number of hits against *Citrus sinensis* at 6,886 hits, followed by *Citrus clementina* at 4,687 hits, *Theobroma cacao* at 1,453 hits, *Ricinus communis* at 1,005 hits, *Vitis vinifera* at 755 hits, *Manihot esculenta* at 665 hits, *Jatropha curcas* at 656 hits and *Ziziphus jujuba* at 538 hits (Fig. 3). It depicts that the higher similarity of *Acer truncatum* unigenes and *Citrus sinensis* genes suggests the possibility we can use *Citrus sinensis* transcriptomes and genomes as a reference for further analysis.

**Table 3:** Functional annotation of the *Acer truncatum* transcriptome unigenes.

Database	Unigene	Percentage
NR	71,401	73.57%
Swiss-prot	26,076	26.87%
GO	39,745	40.95%
COG	30,605	31.53%
KEGG	14,708	15.15%
All-annotated	75,499	77.79%

**Figure 4:**
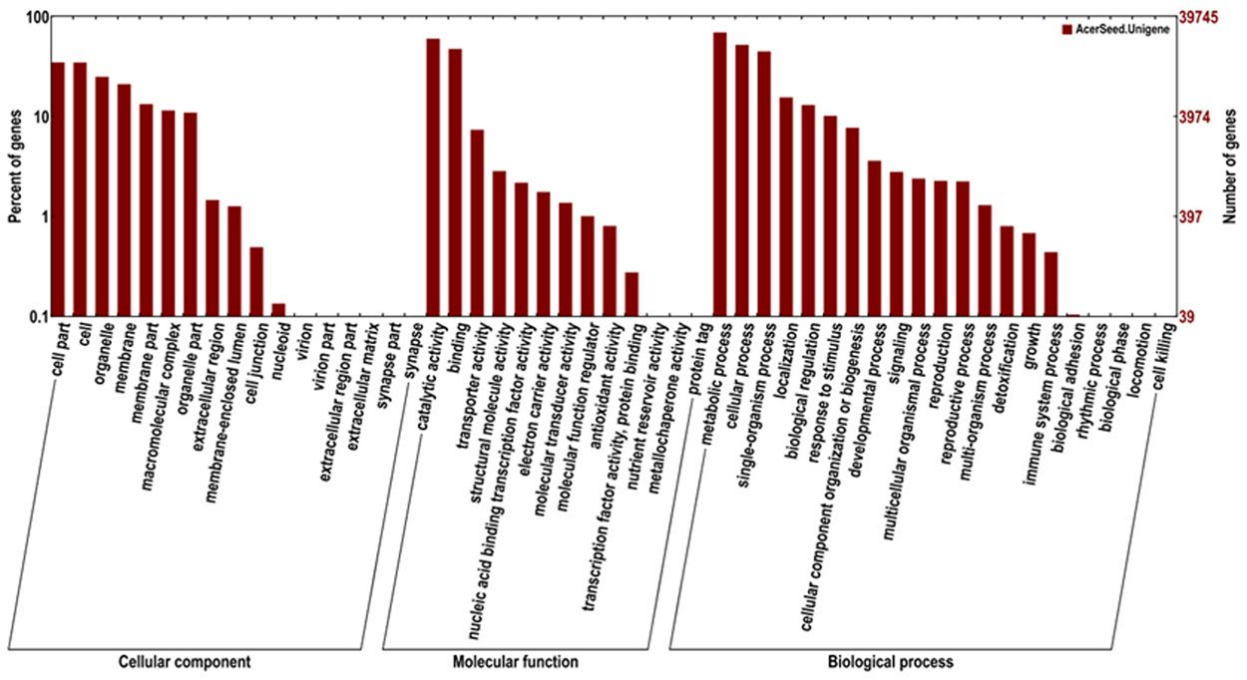
Gene Ontology (GO) classification of assembled unigenes in *Acer truncatum*. The 39,745 *Acer truncatum* unigenes were assigned to three main categories: cellular component, molecular function and biological process.

The SWISS-PROT database is the manually annotated and reviewed protein sequence database. It is a high-quality database of annotated and non-redundant protein sequences, and the results contain experimental results, computational features and scientific conclusions. Among the 97,053 unigenes, 26,076 (26.87%) were similar to the SWISS-PROT database (Table 3). Using the GO database enrichment analysis, the identified assembled unigenes were carried out to classify three independent sets (the cellular component, the molecular function, and the biological process). It depicts that the majority GO terms were assigned to the biological process is 59,615, the molecular function had 27,109 terms assigned, and the cellular component had 60,490 terms assigned (Fig. 4).

**Figure 5:**
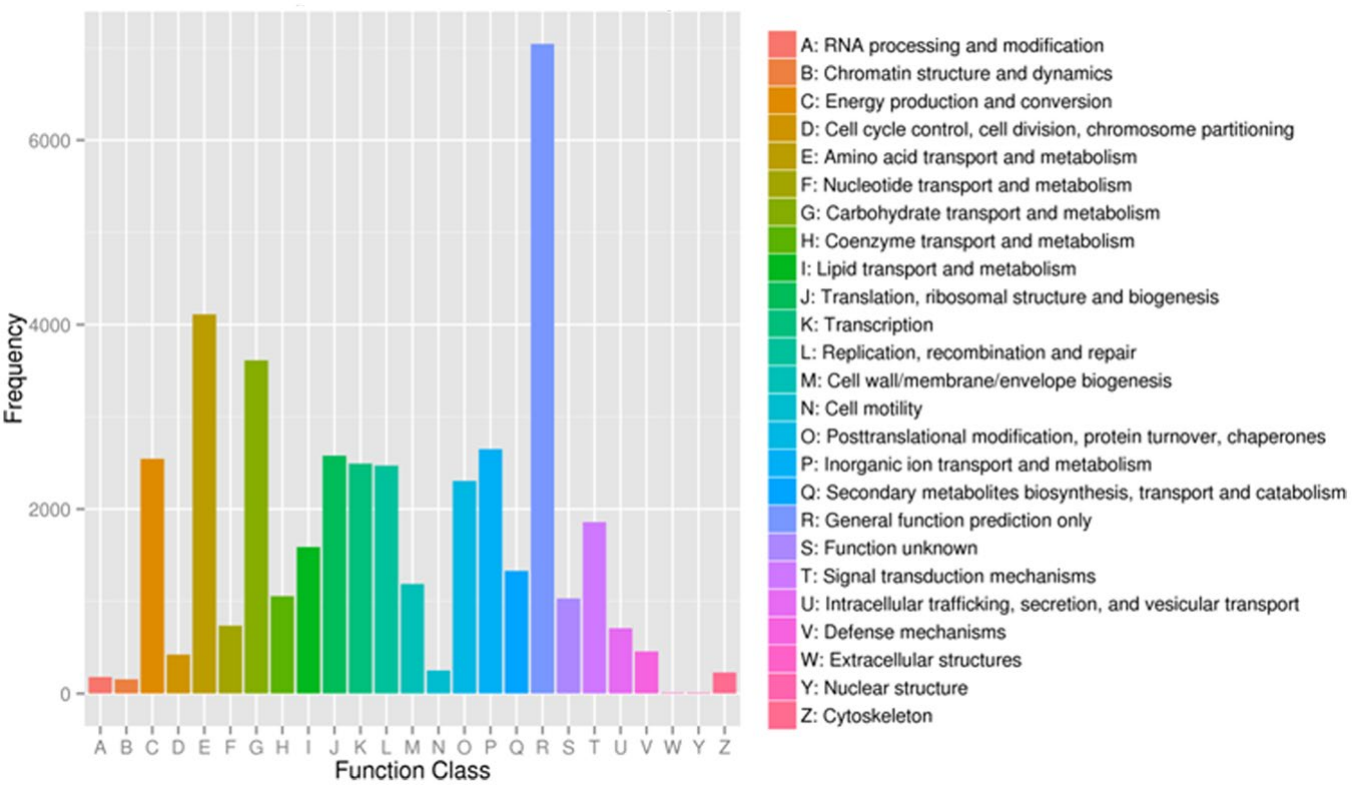
Classification of clusters of orthologous groups (COG). The total of 30,605 *Acer truncatum* unigenes were grouped into 25 classificat.

Furthermore, the assembled unigenes were searched against the COG database. A total of 30,605 unigenes have been assigned to the COG classification (Fig. 5). The highest group is the cluster for function prediction only (7,042, 23.01%), and followed by amino acid transport and metabolism (4,110, 13.43%); carbohydrate transport and metabolism (3,613, 11.81%); inorganic ion transport and metabolism (2,651, 8.66%); translation, ribosomal structure and biogenesis (2,579, 8.43%); energy production and conversion (2,545, 8.32%); transcription (2,493, 8.15%); replication, recombination and repair (2,472, 8.08%); posttranslational modification, protein turnover, chaperones (2,304, 7.53%) and signal transduction mechanisms (1,860, 6.08%). However, only 11 and 10 annotations of unigenes are annotated to the nuclear structural and the extracellular structure. The KEGG database was used to analyze the the active biological pathways. The 14,708 assembled unigenes were assigned to 120 biological pathways through this process (Table S4). Among them, the highest metabolic pathway assigned to the unigenes is Ribosome (ko03010, 698 unigenes), followed by Oxidative phosphorylation (ko00190, 566 unigenes), Purine metabolism (ko00230, 563 unigenes), Protein processing in endoplasmic reticulum (ko04141, 526 unigenes), Glycolysis/Gluconeogenesis (ko00010, 491 unigenes), RNA transport (ko03013, 423 unigenes), Spliceosome (ko03040,414 unigenes) and Pyrimidine metabolism (ko00240, 391 unigenes). These results indicate that the growth and development of *Acer truncatum* seeds is mainly dependent on a large number of substances and energy metabolism.

### EST-SSR discovery

As containing highly information molecular marker, SSR markers have become one of the most widely used molecular marker systems for genetics, evolution and breeding research. To explore EST-SSR markers in the assembled unigenes, the 4,039 sequences containing 5,774 EST-SSRs were produced from 15,837 unigenes. Di-nucleotide and tri-nucleotide motifs were the most plentiful with 31.24% (909) and 29.35% (854), respectively (Table 4). The most repeat was AG/CT (1,012), followed by AGC/CTT (400), AT/AT (328), and ACC/GGT (189) (Table S5). The large set of EST-SSR markers identified in this research will help future researchers to better understand the genome-wide adaptive pattern of this species.

**Table 4:** Length distribution of EST-SSRs based on the number of repeat units.

Number of repeat units	Di-	Tri-	Tetra-	Penta-	Hexa-	Total	Percentage
5	—	856	41	8	4	909	31.24%
6	454	383	11	2	4	854	29.35%
7	297	195	1	1	0	494	16.98%
8	224	20	1	0	0	245	8.42%
9	200	0	0	0	0	200	6.87%
10	189	10	0	0	0	199	6.84%
>10	7	1	1	0	0	9	0.31%

### Candidate enzymes involved in VLCFAs elongation of fatty acids in *Acer truncatum* seeds

VLCFAs are synthesized by a microsomal fatty acid extension (FAE) system. Specifically, the C18 fatty acid is first synthesized using the de novo fatty acid synthesis pathway of plastids, and the C2 portion of malonyl-coenzyme A (CoA) is sequentially added to the previously synthesized C18 fatty acids^8,9^. Analogous to FAS, the elongase complex catalyzes (FAE) is a membrane-bound fatty acid elongation complex, which involves four VLCFAs elongase complex catalyzes: KCS, KCR, HCD, and ECR^10^.

**Figure 6:**
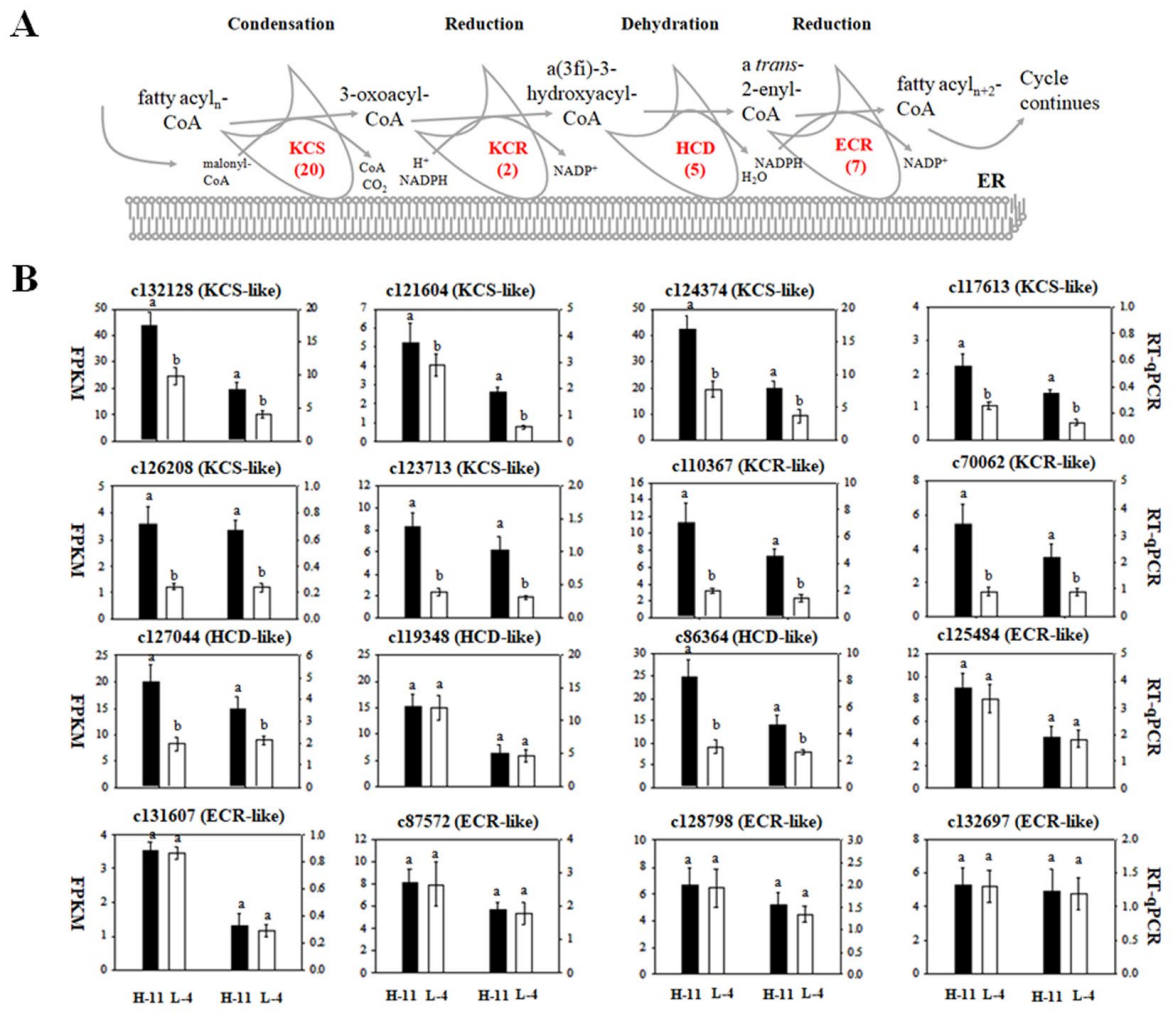
*Acer truncatum* unigenes that may be involved in the fatty acid elongation. (**A**) The fatty acid elongase complex. (**B**) The expression level of DEGs related fatty acid elongation in the “H-11” and “L-4”. Error bars are standard errors of the mean from three technical replicates. The different letters indicates significant differences at P < 0.05 (Student’s t-test).

Based on previous studies, 34 assembled unigenes related to 4 of the enzymes in the VLCFAs FAE were identified in the annotated *Acer truncatum* seeds transcriptome database (Fig. 6A). In the transcriptome database, *KCS* is considered to be the rate-limiting enzyme in VLCFAs biosynthesis. Therefore, the isolation and functional analyses of the genes of *KCS* genes have become the most important thing in the study of VLCFAs biosynthesis. Twenty unigene sequences were annotated as encoding *KCS* genes.

In order to identify the key genes regulating the VLCFAs biosynthesis, DEGs analysis was performed through comparing the expression levels. On the basis of the applied thresholds FDR (False Discovery Rate) <0.01 and log2 (foldchange) ≥2, a total of 3,258 unigenes were identified as DEGs between these two samples (H-11 vs. L-4), which comprised 2,131 up-regulated genes and 1,127 down-regulated unigenes (Fig. S2). Among the 3,258 DEGs, the expression profile of 4 differentially expressed genes in VLCFAs elongation of fatty acids such as *KCS*, *KCR*, *HCD*, and *ECR* gene were DEGs analysis and confirm the results by RT-qPCR (Fig. 6B). The higher content of VLCFAs genotype group (H-28, H-7, H-14, H-26) and the lower content of VLCFAs genotype group (L-1, L-18, L-19) in 36 genotypes were also DEGs analysis and confirm the results by RT-qPCR (Fig. S3).

The results showed that 16 DEGs analysis exhibited the similar trends with the RT-qPCR transcript levels, among them, 10 DEGs were up-regulated, including c132128 (KCS-like), c121604 (KCS-like), c124374 (KCS-like), c117613 (KCS-like), c126208 (KCS-like), c123713 (KCS-like), c110367 (KCR-like), c70062 (KCR-like), c127044 (HCD-like), c86364 (HCD-like) and 6 DEGs showed no difference between H-11 and L-4, sunch as c119348 (HCD-like), c125484 (ECR-like), c87572 (ECR-like), c128798 (ECR-like), c132697 (ECR-like) and c131607 (ECR-like) (Fig. 6B). These results again confirm that KCS is the rate-limiting enzyme for the elongation of long-chain fatty acids. In addition, regulating the expression of the *KCS* gene affects the final contents of VLCFAs. However, since VLCFAs biosynthesis are complex processes involving multiple parameters, we only clarifies part of the whole process through the transcriptome sequencing analysis, so it is difficult to determine a precise conclusion. Obviously, additional accurate molecular biology, genomics and proteomic analysis procedures studies are required to verify and validate and further build on our predictions.

## Conclusions and Perspectives

To our knowledge, this study provides the first comprehensive of the transcriptome of *Acer truncatum* seeds. The coverage of the transcriptome, which includes 37.07 Gbp, was comprehensive enough to identify the majority of the regulation VLCFAs biosynthesis genes. A saturation curve for RNA-Seq was provided in Fig. S4. A total of 127,791 different transcripts and 97,053 unigenes were identified in this study. Additionally, we categorized the enzymes involved in the biosynthesis of VLCFAs, such as KCS, KCR, HCD and ECR. Additionally, large numbers of EST-SSRs were determined. This transcriptome dataset have been made publicly available NCBI Short Read Archive (Accession Number: SUB3838977), we believe that it may provide new resource for future high-throughput gene expression of *Acer truncatum* seeds growth and development as well as its breeding, especially involved in VLCFAs biosynthesis.

## Materials and Methods

### Plant materials

Thirty-six genotypes of *Acer truncatum* trees (10-year-old) seeds, were randomly collected from the Fufeng commercial planting base in Boji Country (N34°22′36.62″; E107°53′44.37″; with altitude 550–600 m), Shannxi Province, China on the early September, 2015. The *Acer truncatum* trees seeds were fully mature in October. The reason for choosing this period is as follows: Our team previously found that VLCFA (especially 24:1 nervonic acid) are converted and accumulated in the middle and late stages of fatty acid synthesis, which is the first of the *Acer truncatum* seeds rich in VLCFA nervonic acid^3^.

### Fatty acids extraction and composition analysis

The seeds of *Acer truncatum*, with three biological replicates, were used for extraction of fatty acids. Seeds FA were extracted and analyzed as reported previously in detail^3^. Briefly, total FA was converted to FA methyl ester at 80 °C in a methanol solution containing 1 M HCl for 2 hours. After extraction, the fatty acid composition of the oil was analyzed by gas chromatography-mass spectrometry (SQ GC-MS, Thermo Fisher). The FAME peaks were identified using the NIST 2014 database and their retention times compared to real standards. Prior to data analysis and statistics, all FAME peaks were quantified by area normalization with a threshold set at 0.1%.

### The RNA-seq library construction for sequencing

The seeds were collected from *Acer truncatum* plants and dissected. After removal of pericarp, they were then immediately frozen and stored in liquid nitrogen prior to further analysis. We extracted the total mRNA using the Plant-RNA Kit (Aidlab -biotech, China). The spectrophotometer and the Agilent 2100 bioanalyzer were used to measure the quality and quantity of purified mRNA. The mRNA-seq library was constructed using the Illumina’s TruSeq RNA Sample Preparation Kit and the library quality was assessed on the Agilent Bioanalyzer 2100 system as previously reported in detail^27^. The fragment (340 bp ± 25 bp) was purified by gel electrophoresis and then amplified by PCR as a sequencing template. Finally, the mRNA library was sequenced by the HiSeq^TM^ 4000 platform (Illumina Inc., USA).

### Illumina sequencing data analysis and assembly

To obtain high quality clean read data for *de novo* assembly, all adapter sequences and low quality sequences were removed from the raw data. Using the Trinity program (k-mer = 25), the high-quality reads were *de novo* assembled (http://trinityrnaseq.sourceforge.net/)^28^. The contigs were clustered and the transcripts were further assembled according to the pairend reads. The longest transcript in the cluster were defined as unigenes. We used EMBOSS Getorf Software to predict the coding area (http://emboss.bioinformatics.nl/cgi-bin/emboss/getorf).

### Sequence clustering and functional categorization of unigenes

The assembled unigenes sequences were annotated based on the following databases: NR (NCBI non-redundant protein sequences); NT (nonredundant nucleotide sequence); the Swiss-Prot (Protein sequence database); GO (Gene Ontology); COG (Clusters of Orthologous Groups of proteins) and KEGG (Kyoto Encyclopedia of Genes and Genomes). The best alignment was selected from the matches with an E-value ≤ 10^−5^. According to the best BLAST comparison (highest score), we give a gene name for each assembly sequence. The ORFs were identified by the “GetORF” (EMBOSS software package)^32,33^. GO were assigned to the assembled unigene using the “Blast2GO”^34,35^. The KEGG pathways annotation by the KAAS (KEGG Automatic Annotation Server) (http://www.genome.jp/kegg/kaas/)^36^.

### The EST-SSRs detection

Using the MISA software, the 12,845 unigenes (more than 1 kb) of *Acer truncatum* were used for the EST-SSRs detection (http://pgrc.ipk-gatersleben.de/misa/). The parameters were adjusted to identify perfect di-, tri-, tetra-, penta- and hexa-nucleotide motifs with a minimum of 6, 5, 4, 4 and 4 repeats espectively as previously described^37,38^.

### Quantitative real-time reverse transcription PCR (RT-qPCR)

The RT-qPCRs were used to examine expression of potential candidate genes in the VLCFAs biosynthetic pathway in *Acer truncatum* seeds, such as *KCS*, *KCR*, *HCD*, and *ECR*. The expression of these potential candidate genes were calculated by relative quantification with the *actin* house keeping gene as a reference. The specific primers are listed in Table S6 for the RT-qPCR reaction according to the candidate genes. The RT-qPCR reactions were performed in a Step One Plus Real-Time PCR System (Applied Biosystems, USA) using a Super Real PreMix kit (SYBR Green) (Tiangen-biotech, China). The RNA relative expression of each gene was calculated as reported previously in detail^31^.

## Electronic supplementary material

Supplementary information
