# Supplementary material for: Comparative transcriptome analysis two genotypes of Acer truncatum Bunge seeds reveals candidate genes that influences seed VLCFAs accumulation
Source: Sci Rep. 2018 Oct 19;8:15504. doi: 10.1038/s41598-018-33999-3 (PMC6195533; doi:10.1038/s41598-018-33999-3)
Supplement: Supplementary file 1 — Supplementary information [file 41598_2018_33999_MOESM1_ESM.docx]

**Supplementary information**

**Title:** Comparative transcriptome analysis two genotypes of *Acer truncatum* Bunge seeds reveals candidate genes that influences seed VLCFAs accumulation

**Authors and institute or laboratory of origin:**

Rongkai Wang, Pei Liu, Jinshuan Fan, Lingli Li^*^

College of Forestry, Northwest A&F University, Yangling 712100, China

***Correspondence:** Lingli Li, Fax: +86 029 87082256

Email: lilingliabc@163.com; lill@nwsuaf.edu.cn

**Table S1.** Comparison of contents of seed total FA content in 36 genotypes *Acer truncatum* seeds.

**Table S2**. Comparison of contents of major seed VLCFA compositions (%) in 36 genotypes *Acer truncatum* seeds FA.

**Table S3.** Overview of the *Acer truncatum* transcriptome assembly.

**Table S4.** The distribution of pathways annotated in the Kyoto Encyclopedia of Genes and Genomes (KEGG).

**Table S5.** Summary of EST-SSRs types in the *Acer truncatum* transcriptome.

**Table S6.** The primers used for qRT-PCR.

**Figure. S1.** Length distribution of *Acer truncatum* transcripts.

**Figure. S2.** The expression profiles of DEGs between these two samples (H-11 vs. L-4).

**Figure. S3.** The qRT-PCRs of the related fatty acid elongation in the higher content of VLCFAs genotype group (H-28, H-7, H-14, H-26) and the lower genotype group (L-1, L-18, L-19).

**Figure. S4.** A saturation curve for RNA-Seq.

**Table S1.** **Comparison of contents of seed total FA content in 36 genotypes *Acer truncatum* seeds.**

| **Sample** | **Seed total FA (mg/g)** |
| --- | --- |
| **1** | 372.51 |
| **2** | 369.72 |
| **3** | 388.93 |
| **4** | 385.35 |
| **5** | 366.61 |
| **6** | 360.12 |
| **7** | 382.41 |
| **8** | 389.33 |
| **9** | 392.72 |
| **10** | 424.11 |
| **11** | 424.52 |
| **12** | 364.42 |
| **13** | 325.71 |
| **14** | 366.22 |
| **15** | 396.51 |
| **16** | 420.71 |
| **17** | 287.82 |
| **18** | 412.53 |
| **19** | 287.54 |
| **20** | 351.21 |
| **21** | 377.82 |
| **22** | 341.12 |
| **23** | 298.51 |
| **24** | 368.62 |
| **25** | 368.61 |
| **26** | 344.21 |
| **27** | 379.91 |
| **28** | 370.21 |
| **29** | 350.11 |
| **30** | 385.31 |
| **31** | 365.23 |
| **32** | 364.22 |
| **33** | 415.42 |
| **34** | 358.22 |
| **35** | 414.51 |
| **36** | 316.11 |
| *The values shown are analyses seeds from 3 independent measurements. | |

**Table S2. Comparison of contents of major seed VLCFA compositions (%) in 36 genotypes *Acer truncatum* seeds FA.**

| **Sample** | **C20:0** | **C20:1** | **C22:0** | **C22:1** | **C24:0** | **C24:1** | **VLCFAs** |
| --- | --- | --- | --- | --- | --- | --- | --- |
| **1** | 0.12 | 7.69 | 0.78 | 18.15 | 0.27 | 6.15 | 33.16 |
| **2** | 0.10 | 8.20 | 0.78 | 20.25 | 0.44 | 7.36 | 37.13 |
| **3** | 0.18 | 8.97 | 0.82 | 19.65 | 0.36 | 6.39 | 36.37 |
| **4** | 0.11 | 7.22 | 0.7 | 17.08 | 0.23 | 4.75 | 30.09 |
| **5** | 0.14 | 7.15 | 0.79 | 20.23 | 0.62 | 7.52 | 36.45 |
| **6** | 0.11 | 7.32 | 0.81 | 19.51 | 0.29 | 6.49 | 34.53 |
| **7** | 0.14 | 7.26 | 0.79 | 20.92 | 0.41 | 8.75 | 38.27 |
| **8** | 0.06 | 7.17 | 0.62 | 19.86 | 0.31 | 7.71 | 35.73 |
| **9** | 0.07 | 7.1 | 0.62 | 21 | 0.34 | 7.69 | 36.82 |
| **10** | 0.15 | 8.17 | 0.79 | 20.57 | 0.38 | 6.57 | 36.63 |
| **11** | 0.12 | 7.49 | 0.83 | 22.3 | 0.6 | 9.19 | 40.53 |
| **12** | 0.11 | 7.17 | 0.75 | 20.3 | 0.37 | 7.44 | 36.14 |
| **13** | 0.09 | 7.49 | 0.63 | 19.85 | 0.34 | 6.83 | 35.23 |
| **14** | 0.08 | 6.98 | 0.73 | 22.06 | 0.31 | 7.88 | 38.04 |
| **15** | 0.1 | 7.86 | 0.71 | 20.24 | 0.32 | 7.39 | 36.62 |
| **16** | 0.08 | 6.93 | 0.63 | 20.21 | 0.3 | 7.77 | 35.92 |
| **17** | 0.08 | 7.34 | 0.6 | 20.56 | 0.22 | 7.42 | 36.22 |
| **18** | 0.07 | 7.47 | 0.56 | 18.31 | 0.18 | 5.69 | 32.28 |
| **19** | 0.12 | 7.93 | 0.61 | 17.14 | 0.17 | 5.29 | 31.26 |
| **20** | 0.08 | 7.19 | 0.64 | 19.18 | 0.26 | 7.20 | 34.55 |
| **21** | 0.06 | 7.43 | 0.68 | 19.89 | 0.24 | 7.69 | 35.99 |
| **22** | 0.08 | 7.05 | 0.71 | 21.26 | 0.35 | 7.90 | 37.35 |
| **23** | 0.08 | 6.99 | 0.67 | 20.97 | 0.37 | 7.17 | 36.25 |
| **24** | 0.08 | 7.08 | 0.66 | 20.54 | 0.36 | 7.47 | 36.19 |
| **25** | 0.09 | 7.33 | 0.64 | 20.96 | 0.24 | 7.90 | 37.16 |
| **26** | 0.10 | 6.90 | 0.91 | 21.95 | 0.43 | 7.74 | 38.03 |
| **27** | 0.08 | 7.10 | 0.75 | 21.89 | 0.32 | 7.68 | 37.82 |
| **28** | 0.10 | 7.30 | 0.82 | 22.50 | 0.42 | 8.06 | 39.20 |
| **29** | 0.11 | 7.54 | 0.71 | 20.24 | 0.27 | 7.41 | 36.28 |
| **30** | 0.12 | 7.79 | 0.65 | 19.48 | 0.26 | 6.38 | 34.68 |
| **31** | 0.12 | 7.16 | 0.81 | 21.23 | 0.31 | 7.82 | 37.45 |
| **32** | 0.08 | 7.60 | 0.72 | 21.58 | 0.24 | 7.55 | 37.77 |
| **33** | 0.06 | 6.84 | 0.72 | 22.20 | 0.26 | 7.68 | 37.76 |
| **34** | 0.12 | 7.96 | 0.73 | 21.29 | 0.23 | 6.78 | 37.11 |
| **35** | 0.10 | 7.39 | 0.78 | 20.63 | 0.34 | 7.78 | 37.02 |
| **36** | 0.14 | 8.19 | 0.72 | 19.70 | 0.24 | 6.76 | 35.75 |
| *VLCFAs=C20:0+C20:1+C22:0+C22:1+C24:0+C24:1. | | | | |  |  |  |
| The values shown are analyses seeds from 3 independent measurements. | | | | | | |  |

**Table S3. Overview of the *Acer truncatum* transcriptome assembly.**

| **Transcripts length** | **Total number** | **percentage** |
| --- | --- | --- |
| **200-300** | 48,320 | 37.81% |
| **300-500** | 27,914 | 21.84% |
| **500-1000** | 24,989 | 19.55% |
| **1000-2000** | 19,531 | 15.28% |
| **2000+** | 7,037 | 5.51% |
| **Total number** | 127,791 |  |
| **Total length** | 87,761,984 |  |
| **N50 length** | 1,122 |  |

**Table S4. The distribution of pathways annotated in the Kyoto Encyclopedia of Genes and Genomes (KEGG).**

| **Pathway** | **Pathway ID** | **Gene number** |
| --- | --- | --- |
| Ribosome | ko03010 | 698 |
| Oxidative phosphorylation | ko00190 | 566 |
| Purine metabolism | ko00230 | 563 |
| Protein processing in endoplasmic reticulum | ko04141 | 526 |
| Glycolysis / Gluconeogenesis | ko00010 | 491 |
| RNA transport | ko03013 | 423 |
| Spliceosome | ko03040 | 414 |
| Pyrimidine metabolism | ko00240 | 391 |
| Alanine, aspartate and glutamate metabolism | ko00250 | 376 |
| Pyruvate metabolism | ko00620 | 373 |
| Aminoacyl-tRNA biosynthesis | ko00970 | 363 |
| Peroxisome | ko04146 | 336 |
| Starch and sucrose metabolism | ko00500 | 329 |
| Arginine and proline metabolism | ko00330 | 323 |
| Citrate cycle (TCA cycle) | ko00020 | 321 |
| Ribosome biogenesis in eukaryotes | ko03008 | 303 |
| Ubiquitin mediated proteolysis | ko04120 | 292 |
| Carbon fixation in photosynthetic organisms | ko00710 | 285 |
| Amino sugar and nucleotide sugar metabolism | ko00520 | 283 |
| Valine, leucine and isoleucine degradation | ko00280 | 264 |
| Cysteine and methionine metabolism | ko00270 | 262 |
| Glycine, serine and threonine metabolism | ko00260 | 259 |
| Propanoate metabolism | ko00640 | 255 |
| Proteasome | ko03050 | 251 |
| Fatty acid metabolism | ko00071 | 239 |
| RNA degradation | ko03018 | 236 |
| Valine, leucine and isoleucine biosynthesis | ko00290 | 231 |
| mRNA surveillance pathway | ko03015 | 230 |
| Tryptophan metabolism | ko00380 | 229 |
| Pentose phosphate pathway | ko00030 | 225 |
| Glutathione metabolism | ko00480 | 224 |
| Glyoxylate and dicarboxylate metabolism | ko00630 | 211 |
| Tyrosine metabolism | ko00350 | 197 |
| Fructose and mannose metabolism | ko00051 | 193 |
| beta-Alanine metabolism | ko00410 | 193 |
| Glycerophospholipid metabolism | ko00564 | 192 |
| Endocytosis | ko04144 | 190 |
| Galactose metabolism | ko00052 | 187 |
| Butanoate metabolism | ko00650 | 181 |
| Phagosome | ko04145 | 181 |
| Phenylalanine, tyrosine and tryptophan biosynthesis | ko00400 | 169 |
| Nucleotide excision repair | ko03420 | 160 |
| Glycerolipid metabolism | ko00561 | 159 |
| Nitrogen metabolism | ko00910 | 158 |
| Plant hormone signal transduction | ko04075 | 157 |
| Phenylalanine metabolism | ko00360 | 155 |
| Biosynthesis of unsaturated fatty acids | ko01040 | 154 |
| N-Glycan biosynthesis | ko00510 | 153 |
| RNA polymerase | ko03020 | 151 |
| Pentose and glucuronate interconversions | ko00040 | 150 |
| Phenylpropanoid biosynthesis | ko00940 | 149 |
| Inositol phosphate metabolism | ko00562 | 140 |
| Plant-pathogen interaction | ko04626 | 139 |
| Porphyrin and chlorophyll metabolism | ko00860 | 131 |
| DNA replication | ko03030 | 127 |
| Fatty acid biosynthesis | ko00061 | 126 |
| Lysine degradation | ko00310 | 125 |
| Protein export | ko03060 | 125 |
| Pantothenate and CoA biosynthesis | ko00770 | 122 |
| Ascorbate and aldarate metabolism | ko00053 | 111 |
| Histidine metabolism | ko00340 | 111 |
| Sulfur metabolism | ko00920 | 106 |
| Selenocompound metabolism | ko00450 | 103 |
| Terpenoid backbone biosynthesis | ko00900 | 103 |
| One carbon pool by folate | ko00670 | 102 |
| Mismatch repair | ko03430 | 100 |
| Base excision repair | ko03410 | 92 |
| Phosphatidylinositol signaling system | ko04070 | 92 |
| Basal transcription factors | ko03022 | 90 |
| Homologous recombination | ko03440 | 90 |
| Limonene and pinene degradation | ko00903 | 85 |
| Cyanoamino acid metabolism | ko00460 | 80 |
| Tropane, piperidine and pyridine alkaloid biosynthesis | ko00960 | 79 |
| Sphingolipid metabolism | ko00600 | 77 |
| Photosynthesis | ko00195 | 73 |
| Steroid biosynthesis | ko00100 | 71 |
| Ubiquinone and other terpenoid-quinone biosynthesis | ko00130 | 68 |
| Isoquinoline alkaloid biosynthesis | ko00950 | 63 |
| Nicotinate and nicotinamide metabolism | ko00760 | 62 |
| Lysine biosynthesis | ko00300 | 60 |
| Folate biosynthesis | ko00790 | 57 |
| Other glycan degradation | ko00511 | 56 |
| Ether lipid metabolism | ko00565 | 51 |
| SNARE interactions in vesicular transport | ko04130 | 49 |
| Sulfur relay system | ko04122 | 48 |
| Glycosylphosphatidylinositol(GPI)-anchor biosynthesis | ko00563 | 46 |
| Taurine and hypotaurine metabolism | ko00430 | 45 |
| Natural killer cell mediated cytotoxicity | ko04650 | 44 |
| Arachidonic acid metabolism | ko00590 | 42 |
| alpha-Linolenic acid metabolism | ko00592 | 42 |
| Thiamine metabolism | ko00730 | 41 |
| ABC transporters | ko02010 | 41 |
| Regulation of autophagy | ko04140 | 41 |
| Carotenoid biosynthesis | ko00906 | 39 |
| Riboflavin metabolism | ko00740 | 37 |
| Vitamin B6 metabolism | ko00750 | 34 |
| Circadian rhythm - plant | ko04712 | 33 |
| Synthesis and degradation of ketone bodies | ko00072 | 32 |
| Glycosphingolipid biosynthesis - globo series | ko00603 | 28 |
| Circadian rhythm - mammal | ko04710 | 26 |
| Flavonoid biosynthesis | ko00941 | 24 |
| Non-homologous end-joining | ko03450 | 24 |
| Glycosaminoglycan degradation | ko00531 | 22 |
| Lipoic acid metabolism | ko00785 | 21 |
| Photosynthesis - antenna proteins | ko00196 | 20 |
| C5-Branched dibasic acid metabolism | ko00660 | 19 |
| Biotin metabolism | ko00780 | 19 |
| Fatty acid elongation in mitochondria | ko00062 | 18 |
| Caffeine metabolism | ko00232 | 18 |
| Zeatin biosynthesis | ko00908 | 18 |
| Glycosphingolipid biosynthesis - ganglio series | ko00604 | 15 |
| Stilbenoid, diarylheptanoid and gingerol biosynthesis | ko00945 | 11 |
| Diterpenoid biosynthesis | ko00904 | 9 |
| Other types of O-glycan biosynthesis | ko00514 | 7 |
| Linoleic acid metabolism | ko00591 | 5 |
| Brassinosteroid biosynthesis | ko00905 | 5 |
| Flavone and flavonol biosynthesis | ko00944 | 3 |
| Glucosinolate biosynthesis | ko00966 | 3 |
| Benzoxazinoid biosynthesis | ko00402 | 1 |
| Betalain biosynthesis | ko00965 | 1 |

**Table S5. Summary of EST-SSRs types in the *Acer truncatum* transcriptome.**

| **Repeat motif** | **Number** | **Percentage** |
| --- | --- | --- |
| **Di-nucleotide** |  |  |
| AC/GT | 105 |  |
| AG/CT | 1012 |  |
| AT/AT | 328 |  |
| CG/CG | 1 |  |
| **Total** | **1446** | **48.59%** |
| **Tri-nucleotide** |  |  |
| AAC/GTT | 155 |  |
| AAG/CTT | 400 |  |
| AAT/ATT | 115 |  |
| ACC/GGT | 189 |  |
| ACG/CGT | 83 |  |
| ACT/AGT | 31 |  |
| AGC/CTG | 143 |  |
| AGG/CCT | 107 |  |
| ATC/ATG | 171 |  |
| CCG/CGG | 62 |  |
| **Total** | **1456** | **48.92%** |
| **Tetra-nucleotide** |  |  |
| AAAC/GTTT | 2 |  |
| AAAG/CTTT | 6 |  |
| AAAT/ATTT | 18 |  |
| AACC/GGTT | 1 |  |
| AACT/AGTT | 1 |  |
| AAGC/CTTG | 3 |  |
| AAGG/CCTT | 1 |  |
| AATC/ATTG | 4 |  |
| AATT/AATT | 1 |  |
| ACAT/ATGT | 2 |  |
| ACTC/AGTG | 7 |  |
| ACTG/AGTC | 2 |  |
| AGAT/ATCT | 2 |  |
| AGCG/CGCT | 2 |  |
| AGGC/CCTG | 1 |  |
| ATCC/ATGG | 1 |  |
| ATCG/ATCG | 1 |  |
| **Total** | **55** | **1.85%** |
| **Penta--nucleotide** |  |  |
| AAAAC/GTTTT | 1 |  |
| AAAAG/CTTTT | 1 |  |
| AAAAT/ATTTT | 1 |  |
| AAATT/AATTT | 1 |  |
| AACAC/GTGTT | 1 |  |
| AAGAG/CTCTT | 1 |  |
| AAGTG/ACTTC | 1 |  |
| AATTC/AATTG | 1 |  |
| ACCAG/CTGGT | 1 |  |
| ACTCC/AGTGG | 1 |  |
| AGAGG/CCTCT | 1 |  |
| **Total** | **11** | **0.37%** |
| **Hexa-nucleotide** |  |  |
| AAAACG/CGTTTT | 1 |  |
| AAAAGC/CTTTTG | 1 |  |
| AAACCG/CGGTTT | 1 |  |
| AATATG/ATATTC | 1 |  |
| ACAGGC/CCTGTG | 1 |  |
| ACCCTG/AGGGTC | 1 |  |
| ACCGCC/CGGTGG | 1 |  |
| ACGAGG/CCTCGT | 1 |  |
| **Total** | **8** | **0.27%** |

**Table S6. The primers used for qRT-PCR.**

| **Gene encoding protein** | **Forward primer** | **Reverse primer** |
| --- | --- | --- |
| c132128 (KCS-like) | ACATAGAAATTAAAAACCAGAG | GCATACACGCAGGAGGGAGAGC |
| c121604 (KCS-like) | ATGGCTCCGAATCCGAAACCG | AGCGCCTCCGGGAAGTACGTCT |
| c124374 (KCS-like) | ATTGGGAGATGATCAGCTTCC | TAGTGAACCGGCACGAACAAGCTT |
| c117613 (KCS-like) | ATGGATCGTGTTCAGATAGATAAG | AACGAGCCCGAGAGAAACAGC |
| c126208 (KCS-like) | GCATCACTAGTTTGGAGACAT | TTAAAGAGCCACAGGATATCTATC |
| c123713 (KCS-like) | TCATATGCGTTGGTTGTGAGC | ATCTTCATTTGAAACACTTTCC |
| c110367 (KCR-like) | TTGCCACATATAGTGGTACGGT | ATCGGCAAAGCCTTCGCTCACC |
| c70062 (KCR-like) | ATGGAGGCTTTGAAAGGTCAG | TCTGGATATCCACGGGGTATATG |
| c119348 (HCD-like) | CAGAGCATGAAATGGTTGGCAAT | ACTTATCTGCGGCAGAGTTGATGT |
| c86364 (HCD-like) | AGACGATTGAGGTCTACAAAGC | ATCCACGTCTCCTGTTGCATGGA |
| c127044 (HCD-like) | GTCGAATAGTACTTCAGATTTCAAC | TGGCAGCTTTACCTTTATCATG |
| c125484 (ECR-like) | AGCAGCCAAGCCATGGAGATGGA | ATGGGATAGATCACCAGAGGCCCA |
| c131607 (ECR-like) | GGTCAACATGGAGCTTCGATTGCTA | AGATGAACCTCTTCCGGGTCCT |
| c87572 (ECR-like) | TGGAGTCACCATTCAAGGGAG | ATGACACATCGTAAATGTGCC |
| c128798 (ECR-like) | TTACTGAGAGCCAATTGGAAG | ATGACTTGTTTAGGGTGGCTAC |
| c132697 (ECR-like) | CTGTCGCAGCACGCAACAATG | AGCGGCCGAAGTAGATGAGC |
| actin | ATCATGTTTGAGACCTTCAACAC | GATCTTCATGAGGTAGTCAGTCAGGT |

**
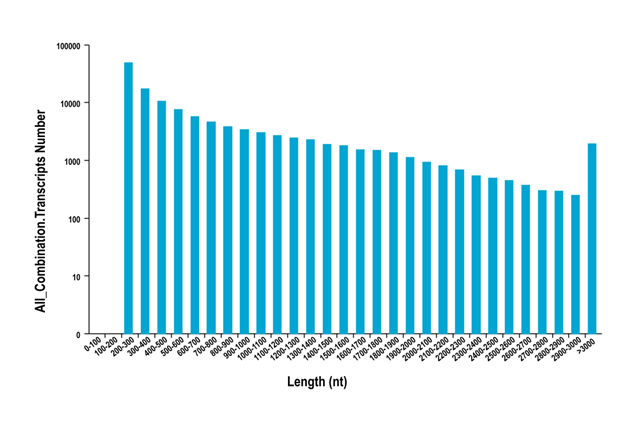
**

**Figure. S1. Length distribution of *Acer truncatum* transcripts.**

**
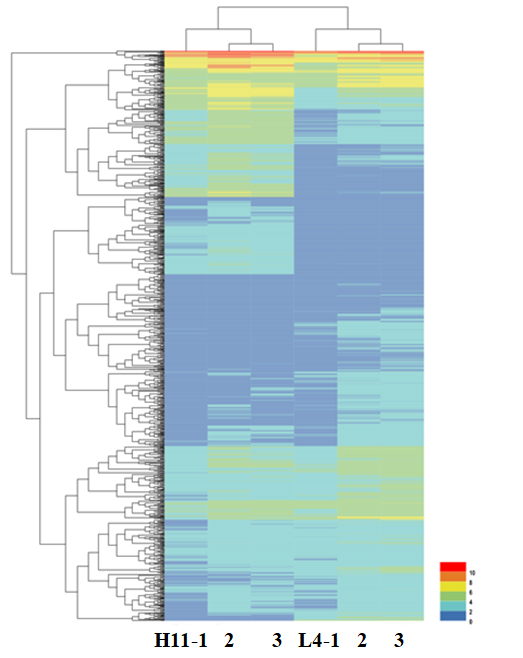
**

**Figure. S2. The expression profiles of DEGs between these two samples (H-11 vs. L-4).**

**
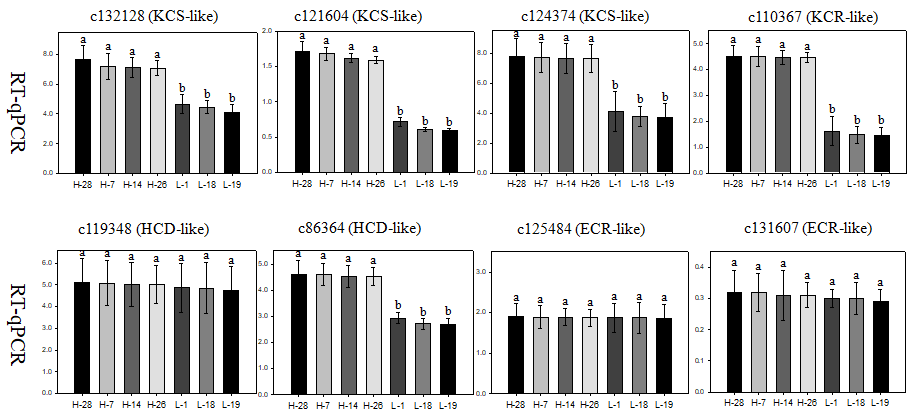
**

**Figure. S3. The qRT-PCRs of the related fatty acid elongation in the higher content of VLCFAs genotype group (H-28, H-7, H-14, H-26) and the lower genotype group (L-1, L-18, L-19).**

**
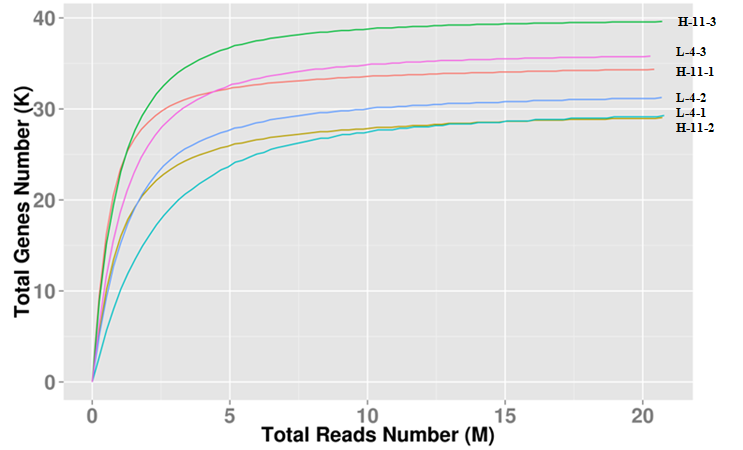
**

**Figure. S4. A saturation curve for RNA-Seq.**
